# Optimizing vacuum drying process of polyphenols, flavanols and DPPH radical scavenging assay in pod husk and bean shell cocoa

**DOI:** 10.1038/s41598-023-40815-0

**Published:** 2023-08-25

**Authors:** Fernando Ramos-Escudero, Sandra Casimiro-Gonzales, María de la Luz Cádiz-Gurrea, Keidy Cancino Chávez, Jaime Basilio-Atencio, Elizabeth S. Ordoñez, Ana María Muñoz, Antonio Segura-Carretero

**Affiliations:** 1 Unidad de Investigación en Nutrición, Salud, Alimentos Funcionales y Nutraceúticos, Universidad San Ignacio de Loyola (UNUSAN-USIL), Calle Toulon 310, 15024 Lima, Peru; 2 Carrera de Nutrición y Dietética, Facultad de Ciencias de la Salud, Universidad San Ignacio de Loyola, Av. La Fontana 550, 15024 Lima, Peru; 3 Instituto de Ciencias de los Alimentos y Nutrición, Universidad San Ignacio de Loyola (ICAN-USIL), Campus Pachacamac, Sección B, Parcela 1, Fundo La Carolina, Pachacámac, 15823 Lima, Peru; 4 Department of Analytical Chemistry, Faculty of Science, University of Granada, Fuentenueva s/n, 18071 Granada, Spain; 5 Facultad de Ingeniería en Industrias Alimentarias, Universidad Nacional Agraria de la Selva, Carretera Central km. 1,2, Tingo María, Peru

**Keywords:** Environmental sciences, Chemistry

## Abstract

The objective of this study was to optimize different vacuum drying conditions for cocoa pod husk and cocoa bean shell in order to enhance these by-products for commercial applications. To carry out the optimization, the response surface methodology was applied using a Box–Behnken experimental design with 15 experiments for which different conditions of temperature (X_1_), drying time (X_2_) and vacuum pressure (X_3_) were established. The response variables were the content of total polyphenols, the content of flavanols and the radical scavenging activity evaluated in the extracts of the different experiments. Temperature (50–70 °C), drying time (3–12 h) and vacuum pressure (50–150 mbar) were considered as independent variables. The main factors affecting the response variables were temperature, followed by vacuum pressure. For the content of polyphenols, the optimal response values predicted for the cocoa pod husk was 11.17 mg GAE/g with a confidence limit (95%) of 9.05 to 13.28 mg GAE/g (optimal conditions: 65 °C, 8 h and 75 mbar), while for the cocoa bean shell cocoa was 29.61 mg GAE/g with a confidence limit (95%) of 26.95 to 32.26 mg GAE/g (optimal conditions: 50 °C, 5 h and 100 mbar). Therefore, results of this study suggest a high content of phenolic compounds obtained from these by-products that show relevance as functional ingredients for application in the food, nutraceutical, and cosmeceutical industries.

## Introduction

Cocoa (*Theobroma cacao* L.) is a plant resource of great economic importance for the main producing regions in the world. Nibs, liquor, cocoa powder, and cocoa butter are obtained during primary processing, while the by-products obtained during pre-processing and processing are cocoa pod husks and cocoa bean shell^1, 2^. The estimated production of cocoa beans (2020/2021) according to the International Cocoa Organization (ICCO) is around 5240 thousand tonnes^3^. Of this production, only one tenth will be used for the production of liquor, butter, cake or cocoa powder, while the remaining biomass (80 to 90%) is discarded as a by-product (including cocoa pod husk, cocoa bean shell, mucilage and placenta)^4^. Cocoa bean shell that are generated during the roasting process represent between 10 and 17% of the total weight of the cocoa bean^5^. The recovery of cocoa by-products from the perspective of the circular economy is essential to promote the value chain and mitigate environmental impacts. From this context, the promotion of innovative models using the cocoa pod husk, and the cocoa bean shell for the production of bioactive components (carbohydrates, dietary fiber, proteins, polysaccharides, polyphenols, minerals, etc.), as well as in the application in food products with high added value (beverages, chocolates, jams, oils, sausages, etc.), and for the production of biofuels (biochar, bioethanol, biogas, bio-oils, etc.) they are highly valued^6–8^.

Several classes of polyphenols have been identified in cocoa pod and cocoa shell, including procyanidins, flavanols, flavonols, phenolic acids^9, 10^. In cocoa shell the main classes of polyphenols are phenolic acids, including gluconic acid, homovanillic acid, vanillic acid glycoside, etc.^10^. These compounds present in cocoa by-products have shown various biological effects^2^. Among the bio-functionalities of the cocoa shell, it is postulated as an antibacterial agent, inhibiting the activity against *Streptococcus mutans*^11^. Rossin et al.^12^ have reported a preventive effect against damage associated with intestinal integrity from oxidative/inflammatory reactions. The results of this study report that probably those responsible for the protection from the adverse effects is its high content of phenolic compounds. Several authors have reported that cocoa shell and pod extracts have antioxidant activity in vitro through DPPH (2,2-diphenyl-1-picrylhydrazyl), ABTS (2,2ʹ-azino-di-(3-ethylbenzothiazoline)) assays -6-sulfonic acid) and FRAP (ferric reducing antioxidant power)^13, 14^. In addition, cocoa shell polyphenols are capable of inhibiting the production of reactive oxygen species, protecting cells from oxidative stress by induction of hydrogen peroxide in human umbilical vein endothelial cells^14^. In the scientific literature, the use of cocoa by-products obtained during pre-processing (cocoa pod) and processing (cocoa shell) has been reported. The approach to the use of these by-products is based on strengthening value chain and the use of its bioactive components as ingredients for functional foods, nutraceuticals, and cosmeceuticals^15^. A previous process for the recovery of bioactive components is the raw material stabilization through different drying conditions such as sun drying, forced air drying oven, vacuum drying, infrared drying, microwave drying, etc.^13^. By-product drying methods present both advantages and disadvantages during the drying process. Water removal from food matrices is a complex process that drastically affects the content of bioactive components, nutrients, and sensory properties, especially the shape, color, aroma, and consistency of dehydrated products^16, 17^. Of most drying methods, forced-air-drying is widely known and widely used as a low-cost method for the industrial production of dehydrated foods from fruits, vegetables, seeds, nuts, and almonds^18^. In addition, the use of conventional technologies during drying has a negative impact on the overall yield and affects the quality of the finished product^19^. On the other hand, vacuum drying is considered an alternative technology compared to conventional methods that use higher temperatures, therefore vacuum drying could promote the conservation of bioactive components present in food^20^. For example, the impact of the drying process on anthocyanins and uncolored phenols in winemaking by-products is highly variable, compared to freeze-drying, which is less drastic for anthocyanins and uncolored phenols^21^. However, freeze-drying processes are not very profitable for the food processing industry due to long times and high process costs^22^. For example, the results of vacuum drying beetroot at 50 °C and 150 mbar on functional properties were comparable to freeze-drying^22^.

The response surface (MRS) methodology is a widely used tool for the optimization process. Some optimized studies based on the vacuum drying process using design of experiments (DOEs) have focused on the evaluation of the physicochemical, functional, antioxidant properties and biochemical changes in order to preserve the bioactive compounds. Šumić et al.^23^ proposed vacuum drying for frozen sour cherries and this method was optimized using a central composite rotatable design (CCRD) to observe the influence of factors (drying temperature and vacuum pressure) on phytochemicals and textural characteristics. Almeida-Trasviña et al.^24^ dehydrated apple pomace in order to optimize the effect of vacuum drying using MRS with a central composite design (CCD). Subsequently, Šumić et al.^25^ dehydrated fresh red currants applying MRS and a Box–Behnken (BBD) experimental design. The optimized conditions for the response variables were established at a vacuum pressure 39 mbar, temperature 70.2 °C and drying time 8 h. Osama et al.^26^ used MRS to maximize vitamin C retention and minimize color degradation and a Box–Behnken design (BBD) was used for vacuum drying experiments. The results of the optimized process after applying MRS were established for the following factors: bed thickness 3.67 mm, vacuum pressure ~ 213.316 mbar, drying temperature 65 °C and blanching temperature 100 °C. The results of these studies are focused on evaluating the effect of vacuum drying to minimize the impact of heat treatment on the response variables.

For this reason, vacuum drying becomes a benchmark for the food industry, its application in food drying is easy and operating costs are lower compared to emerging dehydration technologies, being feasible for small and medium scale processors. Therefore, the objective of the present study was to determine the effects of vacuum drying conditions, such as temperature, drying time, and vacuum pressure, on the content of total polyphenols, flavanols, and DPPH free radical scavenging of cocoa pod husk and cocoa bean shell, by applying a Box–Behnken design (BBD). In addition, moisture, and chromatic properties (L*, a*, and b*) were evaluated.

## Materials and methods

### Plant material

**Figure 1:**
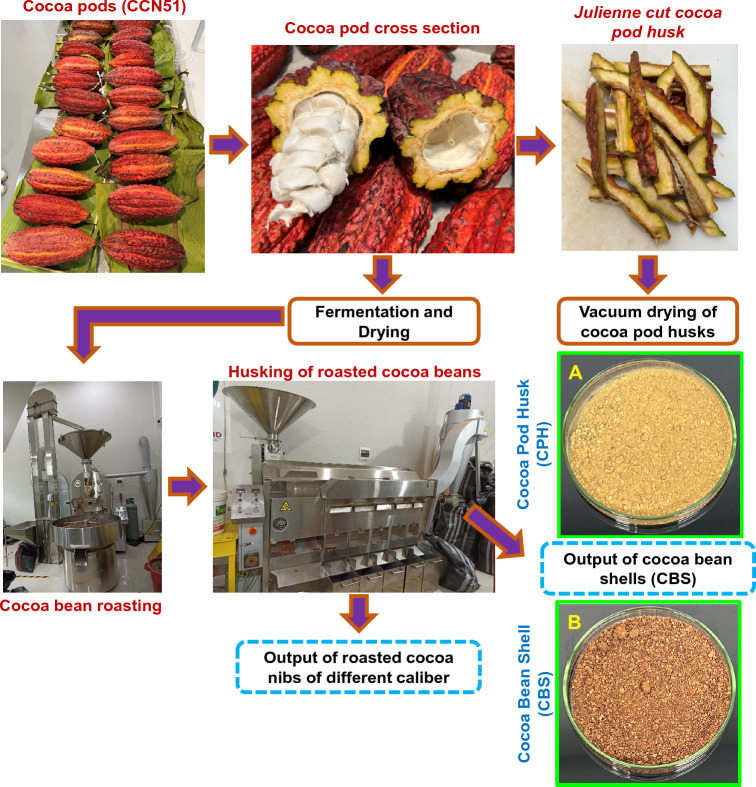
Obtaining cocoa by-products. Cocoa pod husk powder (**A**) and cocoa bean shell powder (**B**).

Cocoa pods (CCN 51) were supplied by the small company Choco Bekita (in the small town of Puerto Ángel, situated in Leoncio Prado province, Peru) in January 2022, while cocoa beans were provided by Cooperativa Agroindustrial Cacao Alto Huallaga (from the Castillo Grande district, Leoncio Prado province, Peru) in November 2021. For this study, the identification of the cocoa fruit (CCN 51 genotype) was not carried out, since it is a genotype widely distributed in different cocoa-producing regions in Peru, as well as in Ecuador, Colombia, and Brazil^27^. Harvest and post-harvest processing was carried out in accordance with the standards established by the International Cocoa Organization (ICCO) (https://www.icco.org/harvesting-post-harvest-new/). The cocoa beans were previously roasted in a toaster oven (IMSA, model ERTC, Oxapampa, Peru). Cocoa shells and nibs were obtained using dehusking and bean grinding machines with an independent outlet for the shells and nibs (IMSA, Oxapampa, Peru) (Fig. 1).

### Cocoa pod pretreatment and vacuum drying

Before the cocoa pod drying process, a cross-section was made for extracting the kernel and placenta. The cocoa pod husk (CPH) was cut into small cubes, placed in bags, and vacuum sealed. The dehydration process was conducted in a vacuum oven (VO 400, Memmert, Schwabach, Germany), and drying processes were performed at different temperatures (50–70 °C), drying times (3–12 h), and pressures (50–150 mbar) as per the Box–Behnken design (BBD) shown in Table 1. The temperature and pressure conditions were the same for the cocoa pods and shells, whereas the drying times varied depending on the sample, CBS (3–5 h), while CPH (8–12 h).

**Table 1:** Factors and levels for cocoa pod husk and cocoa bean shell.

Factors	Symbol	Sample	Levels		
			Low (− 1)	Intermediate (0)	High (1)
Temperature (°C)	X_1_	CPH	50	60	70
		CBS	50	60	70
Time (h)	X_2_	CPH	8	10	12
		CBS	3	4	5
Pressure (mbar)	X_3_	CPH	50	100	150
		CBS	50	100	150

### Moisture content

The moisture content was assessed using a forced air heater (UF160, Memmert, Schwabach, Germany). Approximately 2 g of sample was placed in a Petri dish and dehydrated at 100 °C to constant weight.

### Chromatic properties

Before measuring the chromatic properties, the sample was pulverized using a blade mill (GM 200, Retsch, Haan, Germany). Approximately 500 mg of sample was placed on a glass side and then taken to the adapter base. For color measuring purposes, a Nix QC color sensor (D65 illuminant, 10° observer scan settings, 380–730 spectral range, and measurement geometry of 45°/0°) was used, controlled through a mobile application. Information regarding chromaticity coordinates (L*, a*, and b*) was sent via Bluetooth using the iOS app Nix QC™ installed in an iPhone 13 Pro Max device. The mean color was obtained from 10 scans per sample. Color charts were obtained from https://www.nixsensor.com/free-color-converter/.

### Total polyphenol content (TPC)

Despite Folin–Ciocalteu assay has been widely used to quantify total polyphenols in plant extracts and other food matrices^28^, the mechanism associated of the reaction is not specific, because can react with phenols and non-phenolic compounds generating an overestimating in TPC, despite this evidence the AOAC official method 2017.13 considers it a first action method^29^. Taking into account the previous description, the TPC was estimated using the Folin–Ciocalteu method^30^, with slight modifications. The polar fraction was extracted using approximately 70 mg pod and 50 mg husk, which were placed in 2.0 mL microcentrifuge tubes. Then, 1.0 mL of 50% ethanol solution was added to the mixture and subsequently vortex-agitated (LP Mixer, Thermo Scientific™, South Korea) at 2000 rpm for 1 h. Next, the tubes were placed in an ultrasonic bath (CPX5800HE, Bransonic, Danbury, CT, USA) at a frequency of 40 kHz for an extraction time of 30 min at 30 °C. Finally, the supernatant was obtained using a microcentrifuge (5425R, Eppendorf, Hamburg, Germany) operated at 10,000 rpm for 5 min at 20 °C. Then, 40 μL of a 50% ethanol solution was added to an aliquot of the supernatant (10 μL), followed by adding 375 μL of the Folin–Ciocalteau solution (0.2 N). After a reaction time of 5 min, 375 μL of 7.5% sodium carbonate were added to the mixture. The reaction took 2 h under dark conditions. The absorbance readings were taken in a glass cell for a path length of 2 mm at a wavelength of 765 nm using a UV–Vis spectrophotometer (V-770, Jasco, Tokyo, Japan). The TPC in the sample was expressed in terms of mg of gallic acid equivalent per g of sample (mg GAE/g).

### Flavanol content (TFC)

The flavanol content was measured using the method described by Gallego et al.^31^, modified by Ramos-Escudero et al.^30^. Thus, 200 μL of a 50% ethanol solution was added to an aliquot of the preceding supernatant (25 μL), followed by adding a p-dimethylaminocinnamaldehyde solution, prepared using 0.1% of ethanol solution containing 1 N of hydrochloric acid. The mixture was vortex-agitated for 30 s before being allowed to react for 10 min at room temperature. The absorbance reading was measured at 640 nm using a UV–Vis spectrophotometer (V-770, Jasco, Tokyo, Japan), and the flavanol content was expressed in mg of catechin equivalent per g of sample (mg CE/g).

### DPPH free radical scavenging activity (RSA)

The DPPH test was measured following the method previously described by Brand-Williams et al.^32^, with slight modifications. An aliquot of the previously diluted supernatant (5.0 μL of the extract and 45 μL of 50% ethanol) was mixed with 1.0 mL of the 2.2-diphenyl-1-picrylhydrazyl ([DPPH]; 100 μmol/L in 65% ethanol). The reaction was vortex-agitated for 30 s, after which it was maintained for 10 min at room temperature. Absorbance values were recorded at 515 nm using a UV–Vis spectrophotometer (V-770, Jasco, Tokyo, Japan). The RSA was expressed in mmol of Trolox equivalent per g of sample (mmol TE/g).

### Analysis of methylxanthines and catechin derivatives

Methylxanthines and catechin derivatives of CPH and cocoa bean shell (CBS) were analyzed using a high-performance liquid chromatograph with diode array detector (Hitachi Chromaster™, High-Technologies Corporation, Tokyo, Japan). The analytes were separated using a LiChroCART®150 × 4.6 mm with Purospher® STAR RP-18 endcapped, 5 µm (Burlington, MA), at 30 °C and a flow rate of 1.0 mL/min. Separations were performed in a gradient system using 0.1% orthophosphoric acid in water (mobile phase A) and 0.1% orthophosphoric acid in methanol (mobile phase B)^33^. The gradient program was as follows: 0 min, (20% B); 0–20 min, 40% (B); 20–30 min, 50% (B); 30–32 min, (20% B). The concentration of each component was calculated from the retention times of the respective standards (green tea catechin mix, namely, caffeine, (+)-catechin, (−)-catechin 3-gallate, (−)-epicatechin, (−)-epicatechin-3-gallate, (−)-epigallocatechin 3-gallate, (−)-gallocatechin and (−)-gallocatechin 3-gallate).

### Experimental design and statistical analysis

A BBD was used to optimize the vacuum drying conditions through the response surface methodology (RSM). In this study, the drying condition parameters were temperatures (X_1_, °C), time (X_2_, h), and pressure (X_3_, mbar) and the response variables were TPC, total flavanol content (TFC), and antioxidant activity (AA). A 3-factor and 3-level (3^k^) BBD was used to determine the main influences, as well as the combined ones, for the vacuum drying conditions on the response variables in cocoa pods, and cocoa shell. Furthermore, mathematical models were established between the dehydration factors and response variables (TPC, TFC, and RSA). The design consisted of fifteen experiments, including three focal points that were assigned according to the second-order BBD^34^, with three independent variables. The order in which experiments were conducted was completely random to protect against any unexplained variation effects in the actual responses.

The responses to variables were adjusted to the second-order polynomial model, which describes the interaction between the factors and response variables obtained through RSM, according to the following Eq. (1):

1 $$Y= {\beta }_{0}+ \sum_{i=1}^{k}{\beta }_{i}{x}_{i}+ \sum_{i=1}^{k}{\beta }_{ii}{x}_{i}^{2}+ \sum_{i=1}^{k-1}\sum_{j=2}^{k}{\beta }_{ij}{x}_{i}{x}_{j},$$

where $Y$ is the predicted variable; ${\beta }_{0}$ is a coefficient of the models; ${\beta }_{i}$, ${\beta }_{ii}$, and ${\beta }_{ij}$ are the coefficients of the equations representing the effects of linear, quadratic, and interaction models, respectively; and ${x}_{i}{x}_{j}$ are the independent variables that determine the change generated in the response variable^35^. Statistical analysis was performed using STATISTICA version 8.0 (StatSoft, Inc., Tulsa, OK, USA). Analysis of variance (ANOVA) was performed for each response, with a significance level of p < 0.05, followed by Tukey’s post hoc test. The experiments were conducted in triplicate.

## Results and discussion

### Response surface analysis and CPH optimization

The experimental results of the response values for moisture content, chromatic parameters (L*, a*, and b*), TPC, TFC, and RSA obtained through different vacuum dehydration conditions (temperature, drying time, and pressure) under a BBD are summarized in Table 2. Experiments were conducted after randomization, and each response variable corresponds to the mean value of three repetitions. Before applying the experimental design analysis, the means of different experiments were analyzed using ANOVA, followed by Tukey’s HSD test, to identify the means of experiments that are different.

**Table 2:** Box–Behnken matrix for cocoa pod husk.

Runs	Independent variable								Responses		
	T°	T (h)	mbar	Moisture (%)	Color properties						
	X_1_	X_2_	X_3_		L*	a*	b*	View	TPC	TFC	RSA
1	60	10	100	13.13	58.09	10.64	22.49		10.53 ± 0.47^f^	2.28 ± 0.10^d^	0.11 ± 0.01^b^
2	70	8	100	12.32	61.17	11.05	24.29		11.73 ± 0.63^c^	2.49 ± 0.01^b^	0.12 ± 0.01^a^
3	50	10	50	11.97	59.96	12.72	26.27		8.25 ± 0.02^i^	1.69 ± 0.09^i^	0.08 ± 0.001^e^
4	50	10	150	30.91	35.45	10.03	11.42		2.75 ± 0.31^k^	0.35 ± 0.01^l^	0.01 ± 0.008^h^
5	50	12	100	12.69	58.84	13.11	27.40		8.80 ± 0.34^g^	1.76 ± 0.02^h^	0.08 ± 0.003^e^
6	60	8	150	24.75	40.92	13.55	18.20		4.94 ± 0.64^j^	0.81 ± 0.009^k^	0.04 ± 0.001^g^
7	60	12	50	9.80	64.18	9.66	23.63		11.01 ± 0.35^d^	2.21 ± 0.05^e^	0.10 ± 0.06^b^
8	50	8	100	44.12	33.05	9.17	8.60		1.63 ± 0.03^l^	0.25 ± 0.01^m^	0.003 ± 0.0008^i^
9	60	12	150	11.15	59.51	11.20	24.01		8.33 ± 0.26^hi^	1.69 ± 0.08^i^	0.08 ± 0.0001^e^
10	70	12	100	9.42	63.55	10.72	24.15		10.41 ± 0.10^f^	2.02 ± 0.01^f^	0.09 ± 0.001^c^
11	60	10	100	13.68	54.99	11.15	23.30		12.14 ± 0.05^b^	2.40 ± 0.07^c^	0.12 ± 0.07^a^
12	60	8	50	10.67	62.96	9.82	24.05		13.37 ± 0.11^a^	3.09 ± 0.21^a^	0.12 ± 0.01^a^
13	70	10	150	9.38	57.89	10.01	22.04		8.40 ± 0.04^h^	1.22 ± 0.08^j^	0.06 ± 0.007^f^
14	70	10	50	8.59	63.09	10.87	22.33		10.99 ± 0.08^d^	1.87 ± 0.01^g^	0.09 ± 0.003^d^
15	60	10	100	11.15	57.50	10.03	22.59		10.85 ± 0.04^e^	2.00 ± 0.01^f^	0.09 ± 0.001^c^

TPC (mg GAE/g) total polyphenols; TFC (mg CE/g) total flavanols; RSA (mmol TE/g) by DPPH assay. Different letters by column indicate significant differences at p < 0.05.

The results showed that high temperatures may degrade phenol compounds^36^, with 70 °C being the highest temperature both for the cocoa pod and husk. The TPC of the CPH varied from 1.63 to 13.37 mg GAE/g of sample, whereas the RSA, as measured by the DPPH test, ranged from 0.003 to 0.12 mmol TE/g (Table 2). The results showed that the temperature, drying time, and vacuum pressure parameters influenced the response variables. These results were also observed by Vakula et al.^37^ during the vacuum drying of sweet cherry.

The ANOVA results are described in Table 3. The R^2^ value for the models of response variables was 0.9. TPC (0.958), TFC (0.936), and RSA (0.950; as measured by the DPPH test) and the polynomial models applied were obtained from experimental data. Conversely, the statistical significance values for the responses evaluated were lower than 0.05 in all responses. These p-values of < 0.05 indicate that the mathematical models developed were ideal for experimental data. Furthermore, Fisher’s test used to determine non adjustment (Fisher’s lack-of-fit test) yielded p-values > 0.05, suggesting that the quadratic model properly fit the experimental data.

**Table 3:** Analysis of variance (ANOVA) for the content of total polyphenols, total flavanols and the DPPH free radical scavenging (RSA) of the cocoa pod husk.

ANOVA	df	SS	MS	F-value	p-value
TPC					
Model	11	469.43	42.67	12.68	0.010*
Residual	33	20.70	0.63		
Lack of fit	27	16.34	0.61	0.83	0.664^ns^
Pure error	6	4.36	0.73		
Cor. total	44	490.13			
R^2^	0.958				
TFC					
Model	11	24.68	2.24	8.16	0.020*
Residual	33	1.68	0.05		
Lack of fit	27	1.43	0.05	1.29	0.404^ns^
Pure error	6	0.25	0.04		
Cor. total	44	26.36			
R^2^	0.936				
RSA					
Model	11	0.05	0.00	10.57	0.010*
Residual	33	0.00	0.00		
Lack of fit	27	0.00	0.00	0.42	0.943^ns^
Pure error	6	0.00	0.00		
Cor. total	44	0.06			

*Significant at p ≤ 0.05.

^ns^Not significant at p > 0.05.

The regression data and their statistical significance for each response variable are summarized in Table 4. The TPC shows four terms that demonstrate its statistical significance: temperature (X_1_), vacuum pressure (X_3_) with p-values equal to 0.02, while the interaction between temperature and dehydration time (X_1_X_2_) provided p-values of < 0.015. Furthermore, the temperature factor showed a quadratic effect (X_11_) (p < 0.011), which was found to be considerable for the TPC. Similar results were reported by Almeida-Trasviña et al.^24^ for the coefficients of the linear model X_1_, X_2_ as well as for interactions (X_1_X_2_) and (X_2_X_3_), while the temperature and vacuum pressure factors in the quadratic model exhibited a significant effect on the polyphenol content.

**Table 4:** Regression data of the fitted second-order polynomial models for response variables of the cocoa pod husk (CPH).

Term	Total polyphenol content			Flavanol content			DPPH assay		
Regression data	Coeff.	Std. err.	p-value	Coeff.	Std. err.	p-value	Coeff.	Std. err.	p-value
Intercept	8.38	0.34	0.000*	1.61	0.09	0.000*	0.07	0.004	0.000*
Linear									
X_1_	2.51	0.42	0.002*	0.44	0.12	0.013*	0.02	0.005	0.006*
X_2_	0.86	0.42	0.093^ns^	0.13	0.12	0.319^ns^	0.01	0.005	0.131^ns^
X_3_	− 2.40	0.42	0.002*	− 0.59	0.12	0.004*	− 0.03	0.005	0.004*
Interaction									
X_1_X_2_	− 2.12	0.59	0.015*	− 0.49	0.17	0.031*	− 0.03	0.007	0.017*
X_1_X_3_	0.73	0.59	0.269^ns^	0.17	0.17	0.349^ns^	0.01	0.007	0.231^ns^
X_2_X_3_	1.44	0.59	0.057^ns^	0.44	0.17	0.046*	0.01	0.007	0.108^ns^
Quadratic									
X_11_	− 2.42	0.61	0.011*	− 0.63	0.17	0.015*	− 0.03	0.007	0.009*
X_22_	− 0.61	0.61	0.365^ns^	0.03	0.17	0.856^ns^	0.00	0.007	0.546^ns^
X_33_	− 1.15	0.61	0.117^ns^	− 0.31	0.17	0.134^ns^	− 0.02	0.007	0.052^ns^

(X_1_): temperature, (X_2_): drying time, (X_3_): pressure.

*Significant at p ≤ 0.05.

^ns^Not significant at p > 0.05.

As for the TFC, it could be observed that five terms were found to be statistically significant, namely, temperature (X_1_) and vacuum pressure (X_3_), as their p-values were lower than 0.05 (p = 0.013 and p = 0.004). Meanwhile, interaction was influenced by (X_1_X_2_) and (X_2_X_3_), with p-values of 0.031 and 0.046, respectively. The quadratic factor effect (X_11_) was significantly influenced by vacuum pressure, followed by temperature, whereas the dehydration time showed statistically nonsignificant results (p = 0.319). On the other hand, Rebollo-Hernanz et al.^38^ reported that temperature is the main variable contributing to the retrieval of flavanols 37.7% (p < 0.001), whereas dehydration time contributes just 0.1% (p > 0.05). Šumić et al.^25^ reported that the temperature (X_1_) showed significant differences (p < 0.05) during the red currants vacuum drying process for the content of flavonoids and total polyphenols.

The RSA measured using the DPPH method showed that four terms are responsible for its statistical significance. Similar to the TPC, factors X_1_ and X_3_ have a linear effect on the RSA, given their p-values were lower than 0.01, while the interaction between temperature and time (X_1_X_2_) yielded a p-value of 0.017 and, in quadratic terms, temperature (X_11_) yielded a p-value of 0.009; thus, both the interaction and the quadratic model were found to be statistically significant. These results are similar to those found by Rebollo-Hernanz et al.^38^ in their study in terms of the temperature variables (X_1_) (p < 0.001) and (X_11_) (p < 0.01), while the interaction between temperature (X_1_) and dehydration time (X_2_) was statistically nonsignificant, contributing just 2.5%. On the other hand, Šumić et al.^25^ mentioned that the temperature (X_1_) and the vacuum pressure (X_3_) had a significant influence on the inhibition coefficient at 50% (IC_50_) for the radical DPPH.

The regression equations (Eqs. 2, 3, 4) that describe the response variables for dehydration using vacuum drying for CPH are as follows:

2 $$\begin{aligned} {\text{Y}}_{{{\text{TPC}}}} &= - {15}0.{926 } + { 4}.0{7236} \times {\text{X}}_{{1}} - 0.0{242137} \times {\text{X}}_{{1}} \times {\text{X}}_{{1}} + { 8}.{39838} \times {\text{X}}_{{2}} - \, 0.{152}0{49} \times {\text{X}}_{{2}} \times {\text{X}}_{{2}}\\& \quad- 0.{186957} \times {\text{X}}_{{3}} - 0.000{461833} \times {\text{X}}_{{3}} \times {\text{X}}_{{3}} - 0.{1}0{6118} \times {\text{X}}_{{1}} \times {\text{X}}_{{2}} \\&\quad+ \, 0.00{145575} \times {\text{X}}_{{1}} \times {\text{X}}_{{3}} + \, 0.0{143976} \times {\text{X}}_{{2}} \times {\text{X}}_{{3}} , \end{aligned}$$

3 $$\begin{aligned} {\text{Y}}_{{{\text{TFC}}}}& = - {31}.{4613} + {1}.0{1619} \times {\text{X}}_{{1}} - 0.00{632153} \times {\text{X}}_{{1}} \times {\text{X}}_{{1}} + \, 0.{945365} \times {\text{X}}_{{2}} + \, 0.00{832258} \times {\text{X}}_{{1}}\\&\quad \times {\text{X}}_{{1}} - 0.0{5171}0{2} \times {\text{X}}_{{3}} - 0.000{124629} \times {\text{X}}_{{3}} \times {\text{X}}_{{3}} - 0.0{247685} \times {\text{X}}_{{1}} \times {\text{X}}_{{2}} \\&\quad+ \, 0.000{34494} \times {\text{X}}_{{1}} \times {\text{X}}_{{3}} + \, 0.00{439692} \times {\text{X}}_{{2}} \times {\text{X}}_{{3}} , \end{aligned}$$

4 $$\begin{aligned} {\text{Y}}_{{{\text{RSA}}}}& = - {1}.{72726 } + \, 0.0{474875} \times {\text{X}}_{{1}} - 0.000{285559} \times {\text{X}}_{{1}} \times {\text{X}}_{{1}} + \, 0.0{846286} \times {\text{X}}_{{2}} \\&\quad- 0.000{874837} \times {\text{X}}_{{2}} \times {\text{X}}_{{1}} - 0.00{169}0{76} \times {\text{X}}_{{3}} - {7}.{\text{3657e}} - 0{6} \times {\text{X}}_{{3}} \\&\quad\times {\text{X}}_{{3}} - 0.00{128114} \times {\text{X}}_{{1}} \times {\text{X}}_{{2}} + {2}.0{\text{1257e}} - 0{5} \times {\text{X}}_{{1}} \times {\text{X}}_{{3}} + \, 0.000{144524} \times {\text{X}}_{{2}} \times {\text{X}}_{{3}} . \end{aligned}$$

**Figure 2:**
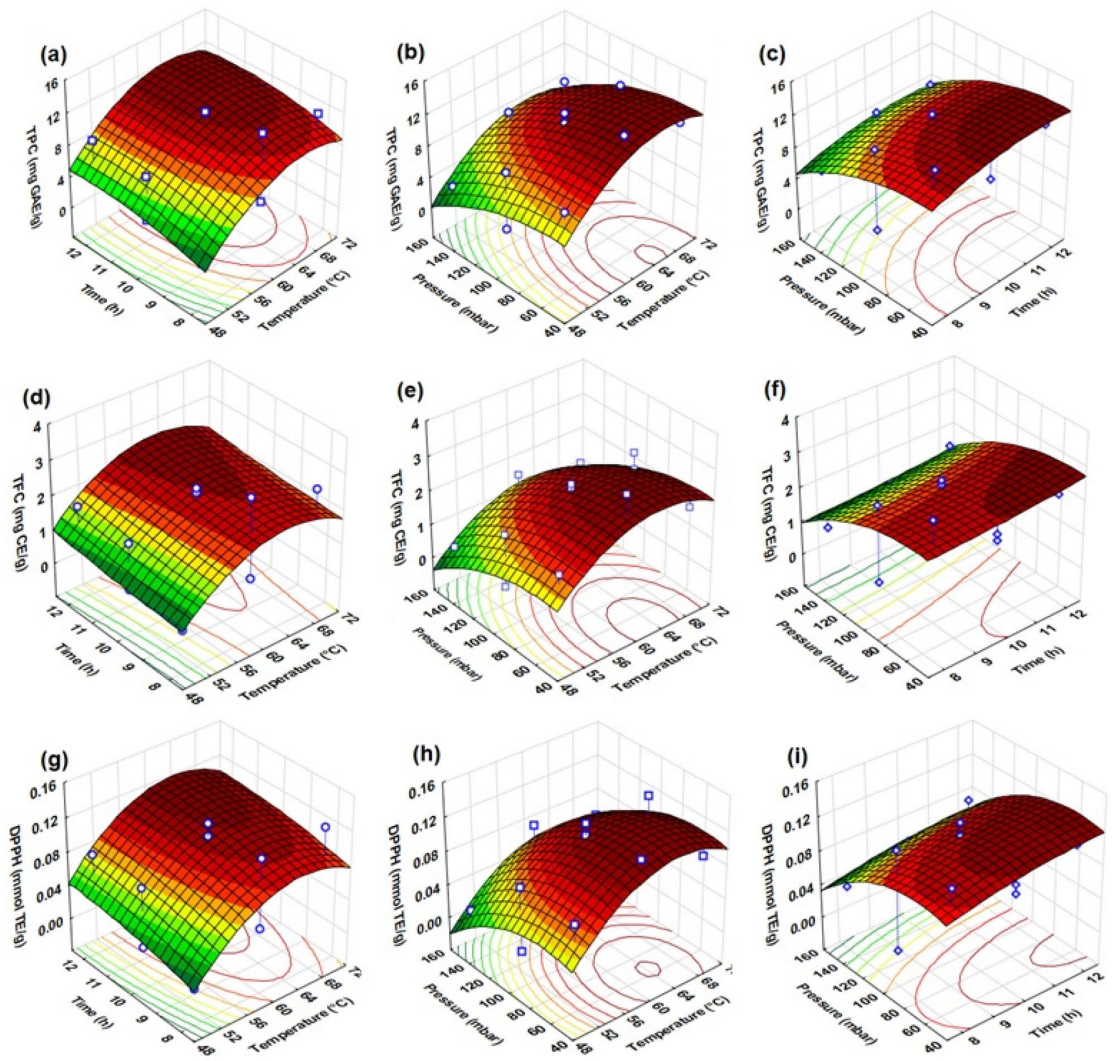
Fitted surface graphs for cocoa pod husk (CPH), showing the combined effects of vacuum drying process of the factor interactions: temperature (°C), drying time (h), and pressure (mbar) for TPC (mg GAE/g) total polyphenols; TFC (mg CE/g) total flavanols; RSA (mmol TE/g) by DPPH assay.

The results for TPC found in this study are very similar to the value reported by Delgado-Ospina et al.^39^, with a mean of 8.44 ± 0.04 mg GAE/g. Another study reported that the TPC ranged from 46 to 57 mg GAE/g, although a low content of it can be found as well. Campos-Vega et al.^40^ reported that the content variation depends on the geographical origin, genotype, and recovery system of bioactive compounds, in addition to the sample’s dehydration method. Table 2 shows that experiment No. 12 had a higher TPC (13.37 mg GAE/g) compared with other experiments. To achieve the maximum polyphenol content, the dehydration process was conducted at 60 °C for 8 h with a vacuum pressure of 50 mbar. On the other hand, experiment No. 8 showed the lowest content of polyphenols, with an average of 1.63 mg GAE/g, under dehydration parameters of 50 °C, 8 h, and 100 mbar. Overall, an increase in the dehydration temperature and time improved the polyphenol content if the vacuum pressure applied was reduced to 50 mbar (Fig. 2a–c). Experiments No. 5, 10, and 7 demonstrated that the TPC increased to 8.80, 10.41, and 11.01 mg GAE/g when pressure decreased from 100 to 50 mbar. These results are similar to those found by Šumić et al.^25^ and Almeida-Trasviña et al.^24^. Therefore, the optimal vacuum drying conditions were the following: temperature (65 °C), drying time (8 h) and vacuum pressure (75 mbar) to obtain a TPC of 11.17 mg GAE/g.

The TFC obtained in the different experiments showed less variation compared to the TPC. Delgado-Ospina et al.^39^ reported that the TFC in CPH was 2.9 ± 0.1 mg rutin equivalent per gram of sample. Table 2 shows that experiment No. 12 had a greater content of flavanols (3.09 mg CE/g), while a content of 0.25 mg CE/g was obtained in experiment No. 8. Both TFC and TPC showed similar results for all experiments (1–15) (correlation coefficient = 0.9415; p = 0.0000). The effect of temperature, drying time, and decreased vacuum pressure play an important role in flavanol retrieval (Fig. 2d–f). Rebollo-Hernanz et al.^38^ also reported that flavanol retrieval improved with an increase in temperature.

**Figure 3:**
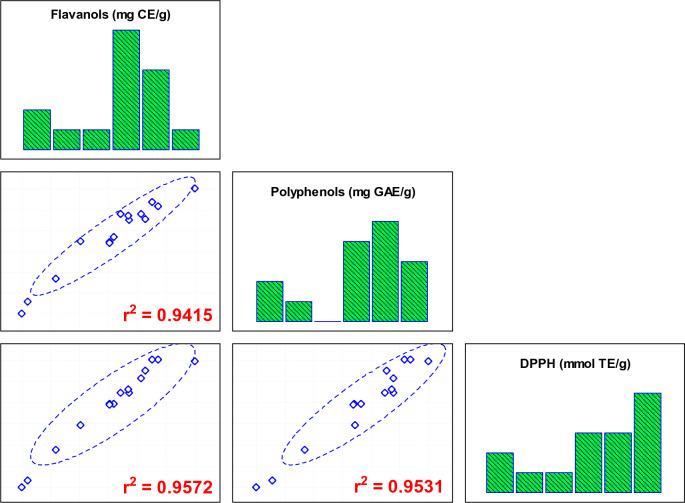
Correlation between response variables for cocoa pod husk: TPC (mg GAE/g) total polyphenols; TFC (mg CE/g) total flavanols; RSA (mmol TE/g) by DPPH assay.

On the other hand, the RSA measured using the DPPH method (Fig. 2g–i) was similar to the mean reported by Delgado-Ospina et al.^39^, with a value of ~ 0.058 mmol TE/g. When 5 categories are formed, the RSA is distributed as follows: in 2 experiments ranging from 0.0035 to 0.0269 mmol TE/g, in 1 experiment ranging from 0.0269 to 0.0503 mmol TE/g, in 1 experiment ranging from 0.0503 to 0.0738 mmol TE/g, in 6 experiments ranging from 0.0738 to 0.0972 mmol TE/g, and in 5 experiments ranging from 0.0972 to 0.1206 mmol TE/g. The first 4 correspond to experiments 4, 6, 8, and 13, with a mean of 0.03 mmol TE/g and an average temperature and vacuum pressure of 57.5 °C and 137.5 mbar, respectively, whereas the 6-experiment group presented a mean of 0.09 mmol TE/g. This group was characterized by an average temperature value of 60 °C and a vacuum pressure of 91.7 mbar. Finally, the last group showed a mean of 0.11 mmol TE/g, with a temperature of 62 °C and a vacuum pressure of 80 mbar. These results are consistent with those reported by Almeida-Trasviña et al.^24^, who found that the best effect for RSA was achieved when the temperature was high, and the vacuum pressure was low. The correlation of DPPH with TPC and with TFC was high: DPPH vs. TPC (r^2^ = 0.9531) and DPPH vs. TFC (r^2^ = 0.9572; Fig. 3). CPH is an excellent source of antioxidants. Indrianingsih et al.^41^ reported that this subproduct contains an important bioactive compound, which has been found to have antioxidant and antidiabetic properties.

### Response surface analysis and CBS optimization

The results obtained with the BBD for the CBS (Table 5) showed that the TPC varied between 19.56 and 35.31 mg GAE/g of sample, the TFC ranged from 5.07 to 6.32 mg CE/g of sample, and the AA ranged from 0.21 to 0.24 mmol TE/g. These results indicate that the polyphenol content was mostly influenced by temperature, drying time, and vacuum pressure factors, compared to the TFC and RSA.

**Table 5:** Box–Behnken matrix for cocoa bean shell.

Runs	Independent variable								Responses		
	T°	t(h)	mbar	Moisture (%)	Color properties						
	X_1_	X_2_	X_3_		L*	a*	b*	View	TPC	TFC	RSA
1	60	5	50	1.48	35.41	11.30	10.07		23.33 ± 0.25^f^	5.12 ± 0.10^c^	0.22 ± 0.02^c^
2	60	3	50	1.89	31.43	9.05	6.14		19.56 ± 0.95^g^	6.27 ± 0.13^a^	0.23 ± 0.01^a^
3	70	5	100	1.02	34.39	11.13	8.55		31.74 ± 0.53^b^	5.56 ± 0.11^b^	0.23 ± 0.01^abc^
4	60	4	100	1.54	32.03	11.43	8.97		29.00 ± 0.30^cd^	6.08 ± 0.11^a^	0.24 ± 0.03^a^
5	60	4	100	1.21	33.73	11.31	9.20		30.84 ± 0.34^b^	5.60 ± 0.07^b^	0.22 ± 0.02^abc^
6	50	4	50	1.28	33.05	11.43	8.61		28.17 ± 0.25^de^	6.32 ± 0.12^a^	0.23 ± 0.01^a^
7	70	4	50	0.74	34.37	13.36	11.22		30.14 ± 0.51^bcd^	5.37 ± 0.08^bc^	0.22 ± 0.01^c^
8	70	4	150	1.43	37.22	8.77	8.04		27.31 ± 0.57^e^	5.68 ± 0.06^b^	0.23 ± 0.03^a^
9	70	3	100	1.31	33.44	8.32	6.55		24.15 ± 0.78^f^	5.07 ± 0.12^c^	0.22 ± ^abcd^
10	60	3	150	1.40	33.78	11.36	10.13		23.46 ± 0.28^f^	6.10 ± 0.09^a^	0.24 ± 0.03^a^
11	50	4	150	1.59	39.69	15.08	16.78		24.47 ± 0.60^f^	5.08 ± 0.07^c^	0.23 ± 0.02^a^
12	50	3	100	2.00	31.91	11.95	8.76		27.60 ± 0.49^de^	5.09 ± 0.09^c^	0.22 ± 0.04^abc^
13	60	5	150	1.04	33.52	12.68	9.99		33.77 ± 0.58^a^	5.61 ± 0.16^b^	0.23 ± 0.02^ab^
14	50	5	100	1.74	33.56	12.56	9.42		35.31 ± 0.57^a^	5.77 ± 0.07^b^	0.23 ± 0.03^a^
15	60	4	100	1.40	36.57	8.96	8.30		28.98 ± 0.50^de^	5.34 ± 0.10^bc^	0.21 ± 0.03^d^

TPC (mg GAE/g) total polyphenols; TFC (mg CE/g) total flavanols; RSA (mmol TE/g) by DPPH assay. Different letters by column indicate significant differences at p < 0.05.

The ANOVA results are summarized in Table 6. As for the shell of cocoa beans, the variables TFC, and RSA were statistically nonsignificant. The F-values for the variables were 0.45, and 0.36, while the p-values were 0.860, and 0.911, respectively. The R^2^ variables for the response variables were far from encouraging, with TPC, TFC, and RSA values of 0.710, 0.449, and 0.393, respectively. Since TPC contribution was significant (p < 0.046), the mathematical model was generated for this variable.

**Table 6:** Analysis of variance (ANOVA) for the content of total polyphenols, total flavanols and the DPPH free radical scavenging (RSA) of the cocoa bean shell.

ANOVA	df	SS	MS	F-value	p-value
TPC					
Model	11	480.77	43.70	88.13	0.000*
Residual	33	298.13	9.03		
Lack of fit	27	244.01	9.04	21.11	0.046*
Pure error	6	54.12	9.02		
Cor. total	44	778.90			
R^2^	0.710				
TFC					
Model	11	6.85	0.23	0.45	0.860^ns^
Residual	33	1.37	0.04		
Lack of fit	27	1.21	0.05	1.74	0.252^ns^
Pure error	6	0.16	0.25		
Cor. total	44	8.22			
R^2^	0.449				
RSA					
Model	11	0.00	0.00	0.36	0.911^ns^
Residual	33	0.00	0.00		
Lack of fit	27	0.00	0.00	0.09	0.999^ns^
Pure error	6	0.00	0.00		
Cor. total	44	0.0027			

*Significant at p ≤ 0.05.

^ns^Not significant at p > 0.05.

The regression equation (Eq. 5) describing the response variable for dehydration through vacuum drying for cocoa bean husk is as follows:

5 $$\begin{aligned} {\text{Y}}_{{{\text{TPC}}}} &= { 55}.{4573 } - { 2}.{97554} \times {\text{X}}_{{1}} + \, 0.0{232}0{52} \times {\text{X}}_{{1}} \times {\text{X}}_{{1}} + { 14}.{8585} \times {\text{X}}_{{2}}\\&\quad - \, 0.{656262} \times {\text{X}}_{{2}} \times {\text{X}}_{{2}} - \, 0.{2598} \times {\text{X}}_{{3}} + \, 0.00{1817}0{9} \times {\text{X}}_{{3}} \times {\text{X}}_{{3}} \\&\quad+ \, 0.00{461714} \times {\text{X}}_{{1}} \times {\text{X}}_{{2}} + \, 0.000{598781} \times {\text{X}}_{{1}} \times {\text{X}}_{{3}} - \, 0.0{163812} \times {\text{X}}_{{2}} \times {\text{X}}_{{3}} . \end{aligned}$$

**Figure 4:**
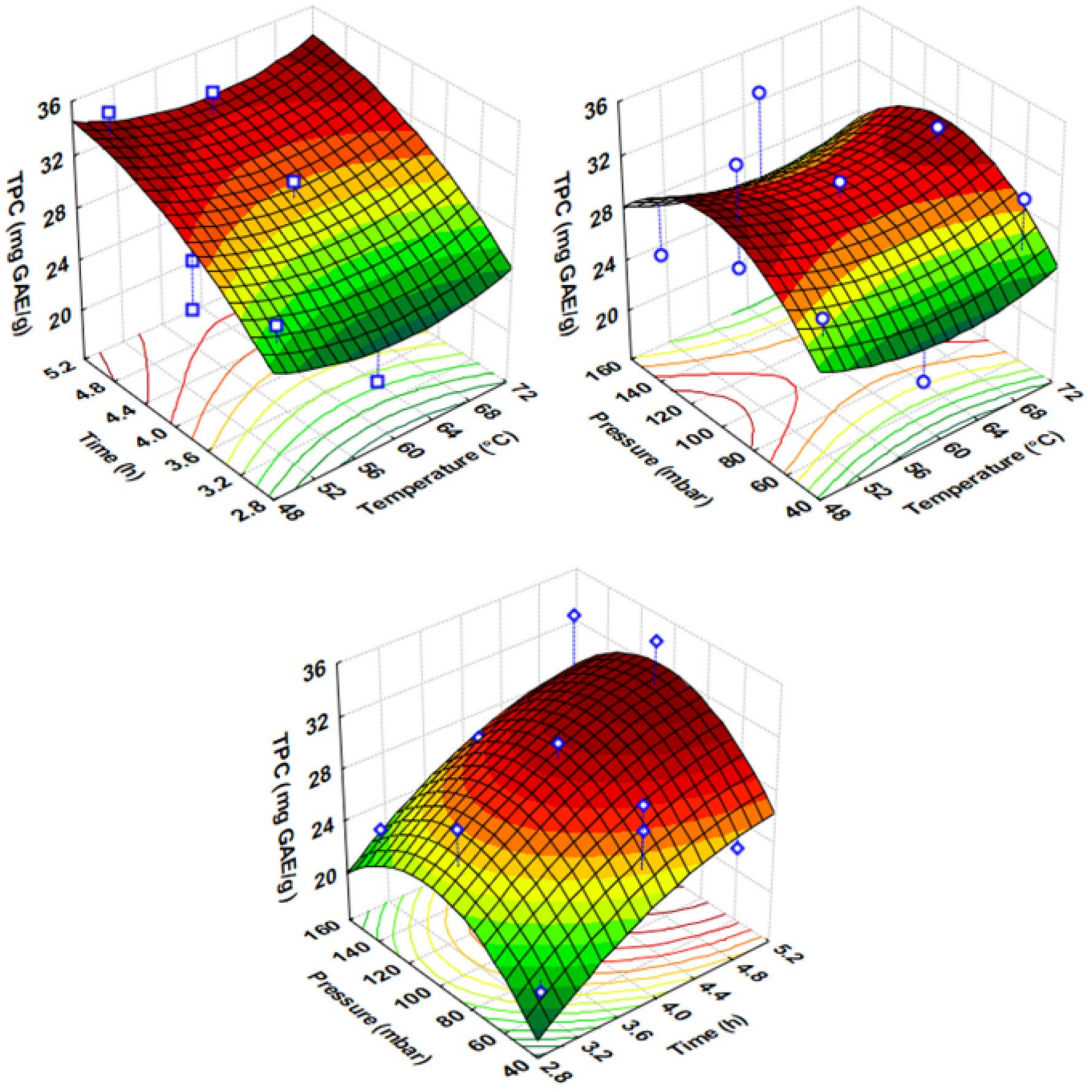
Fitted surface graphs for cocoa bean shell (CBS), showing the combined effects of vacuum drying process of the factor interactions: temperature (°C), drying time (h), and pressure (mbar) for TPC (mg GAE/g).

The representation of the 3D response surface was generated for TPC (Fig. 4), while ANOVA results (Table 7) revealed only one factor as statistically significant for polyphenol content—drying time (X_2_) (p = 0.043). On the other hand, the flavanol content and RSA yielded nonsignificant values (p > 0.05). In addition, the mathematical models generated were not fit to predict the responses. In fact, the lack of fitness was significant for the polyphenol content variable (p < 0.05), while the p-value was 0.271 and 0.826 for the flavanol content and RSA responses, respectively (Table 6). The factors selected did not show a great effect on the response variables but only a slight influence of drying time on polyphenol content in the linear model. On the other hand, vacuum pressure had no significant effect on the responses either. Rebollo-Hernanz et al.^38^ reported a high influence of the temperature factor (X_1_) on polyphenol content, flavanol content, and RSA, with contributions ranging from 37 to 43%. The temperature ranged from 30 to 100 °C during the study. Furthermore, it could be observed that the drying time (X_2_) did not have a significant influence, with a contribution of 0.1–0.5%. The interaction between temperature and drying time (X_1_X_2_) for the three response variables was statistically nonsignificant. As for vacuum pressure (X_3_), Almeida-Trasviña et al.^24^ reported that the effect of X_3_ in the linear model was nonsignificant, both for polyphenol content and RSA.

**Table 7:** Regression data of the fitted second-order polynomial model for response variable (TPC) of the cocoa bean shell (CBS).

Term	Total polyphenol content		
Regression data			
Intercept	27.42	1.11	0.000*
Linear			
X_1_	− 0.28	1.36	0.846^ns^
X_2_	3.67	1.36	0.043*
X_3_	0.98	1.36	0.506^ns^
Interaction			
X_1_X_2_	− 0.03	1.93	0.988^ns^
X_1_X_3_	0.22	1.93	0.915^ns^
X_2_X_3_	1.63	1.93	0.436^ns^
Quadratic			
X_11_	1.29	2.01	0.548^ns^
X_22_	− 1.20	2.01	0.577^ns^
X_33_	− 3.38	2.01	0.153^ns^

(X_1_): temperature, (X_2_): drying time, (X_3_): pressure.

^*^Significant at p ≤ 0.05.

^ns^Not significant at p > 0.05.

The TPC in CBS was quite similar to that reported by Rojo-Poveda et al.^42^, ranging from 3 to 43 mg GAE/g for CBS of different origins—even Peruvian samples had a median of ~ 34.5 mg GAE/g. Conversely, Cádiz-Gurrea et al.^10^ reported bean shell values between ~ 7 and ~ 22 mg GAE/g. These variations in polyphenol content may be associated with geographic origin^43^. Table 5 indicates that experiment No. 14 had a higher polyphenol content, with factors (X_1_ = 50 °C, X_2_ = 5 h, and X_3_ = 100 mbar) statistically nonsignificant compared with experiment No. 13 (X_1_ = 60 °C, X_2_ = 5 h, and X_3_ = 150 mbar). Experiment No. 2 obtained the lowest polyphenol content (19.56 mg GAE/g). Therefore, the optimal vacuum drying conditions were the following: temperature (50 °C), drying time (5 h) and vacuum pressure (100 mbar) to obtain a TPC of 29.61 mg GAE/g.

As for the flavanol content, the experiments performed showed little variation—the lower and upper quartile yielded values ranging from 5.12 to 6.08 mg CE/g, and the difference between the minimum and maximum values was 1.25 mg CE/g of sample. The minimum and maximum values corresponded to experiments No. 9 (X_1_ = 70 °C, X_2_ = 3 h, and X_3_ = 100 mbar) and 6 (X_1_ = 50 °C, X_2_ = 4 h, and X_3_ = 50 mbar), respectively. The mean value of the set of experiments was found in experiment No. 8 (5.68 mg CE/g) (X_1_ = 70 °C, X_2_ = 4 h and X_3_ = 150 mbar). The flavanol content in this study was lower compared to the results found by Cádiz-Gurrea et al.^10^, who reported values from ~ 16 to ~ 36 mg of catechin equivalent per g of sample. These differences may be attributed to geographic origin^42^, sample characteristics, extraction system, and analytical procedure^10^.

The RSA in the experiments varied slightly, with a lower and upper quartile of 0.21 and 0.24 mmol TE/g of sample, respectively. Delgado-Ospina et al.^39^ reported values of ~ 0.071 mmol TE/g, whereas Rojo-Poveda et al.^42^ examined CBS samples of different geographic origins and reported a variation of ~ 0.03 to ~ 0.18 mmol TE/g. Peruvian samples showed a mean of ~ 0.15 mmol TE/g, while the RSA values reported by Barbosa-Pereira et al.^43^ for the CBS of Creole and Trinitarian genotype, native to Venezuela, ranged from ~ 0.017 to ~ 0.026 mmol TE/g. The RSA values obtained in this study were slightly higher than those reported by other researchers^42, 43^.

### Moisture content of CPH and CBS

When the set of experiments was assessed, the moisture content for CPH varied from 8.59 to 44.12% (Table 2). Nguyen et al.^13^ reported moisture values of 9.22%, 10.61%, and 11.99%, with temperatures of 60 °C, 80 °C, and 100 °C for vacuum drying. Three groups can be identified when considering a histogram. The first group, comprising experiments with a moisture range of 5–10%, represented 26.67% of the experiments; the second group, comprising experiments with a moisture range of 10–15%, accounted for 53.33% of the total experiments; and the third group comprised experiments with moisture values > 15%, representing 20% of the experiments. Experiments with low moisture percentages had a mean temperature of 67.50 °C and a mean vacuum pressure of 87.50 mbar, whereas those with a high moisture percentage yielded an average drying temperature value of 53.33 °C and a vacuum pressure of 133.33 mbar. These results were similar to those obtained by Šumić et al.^25^, who reported that an increased vacuum pressure results in slow drying and produces samples with high moisture content. In contrast, when vacuum pressure decreases, the drying process is faster, producing samples with low moisture content. Furthermore, the drying time influenced the moisture content—experiments with values ranging from 5 to 10% yielded an average of 11 h, while those with values ranging from 10 to 15% had a mean of 8.67 h. Moisture content also affected the response variables—experiments with high drying temperatures and low vacuum pressure showed high TPC, TFC, and RSA. Conversely, experiments (4, 6, and 8; Table 2) conducted at low temperature and high vacuum pressure yielded lower values of polyphenol (3.11 mg GAE/g) and flavanol (0.47 mg CE/g) contents as well as an RSA of 0.02 mmol TE/g. Similar results were reported by Almeida-Trasviña et al.^24^, with lower values for TPC and RSA for temperatures ranging from 32 to 41 °C and a vacuum pressure ranging from ~ 420 to ~ 505 mbar.

In the case of CBS, the moisture content varied between 0.74 and 2.0%, which are similar to those published by Delgado-Ospina et al.^39^, who reported a mean of 1.9%. In addition, the moisture content slightly varied as a function of the experiment. However, those between 1.0 and 1.6% accounted for 73.33% of all experiments. This group showed a mean of 53.33 °C, 3.67 h, and a vacuum pressure of 83.33 mbar. Conversely, experiments with a lower moisture percentage showed the following factors: X_1_ = 70 °C, X_2_ = 4 h, and X_3_ = 50 mbar. As in the case of CPH, a decrease in the vacuum pressure provided fairly rapid drying kinetics. For this group of experiments, the TPC was greater than that of experiments with a higher moisture percentage. Nevertheless, the flavanol content and RSA showed no major changes. As for AA, Šumić et al.^25^ found that a higher temperature and vacuum pressure yielded lower values of RSA; thus, high temperatures result in the inactivation of antioxidants.

### Chromatic properties of CPH and CBS

The color parameters of the CPH used in different experiments showed that clarity (L*) varied between 33.05 and 64.18 units (Table 2). Chromaticity coordinates, such as a*, ranged from 9.17 to 13.55 units, while b* varied between 8.60 and 27.40. These chromatic values found for the different experiments are quite similar to those obtained by Delgado-Ospina et al.^39^ and Delgado-Ospina et al.^44^. The variations in color parameters can be attributed to the sample’s stabilization process, such as drying by lyophilization and thermal treatment to inactivate enzymes, followed by lyophilization, sun-drying, vacuum drying, infrared drying, and microwave drying, among others^13^. In this regard, the drying temperatures influenced the chromatic parameters, and experiments that yielded low moisture content values (5–10%) showed clearer L* values (mean: 62.18 units), while experiments with values ranging from 10 to 15% showed slightly lower L* values (mean: 59.13 units). Conversely, much lower L* values were found in experiments with a moisture content exceeding 15% (mean: 36.47 units). These results are very similar to those reported by other authors where the increase in moisture content produces a decrease in the L* parameter. As for the chromaticity parameter a*, no remarkable changes were observed despite the variation in moisture content. Delgado-Ospina et al.^44^ observed similar changes in dehydrated (4.02 units) and hydrated (4.56 units) CPH samples. As for the chromaticity coordinate b*, the different experiments showed considerable variations—treatments with < 15% of moisture showed higher means (approximately 23.67 units), while those with > 15% of moisture yielded a mean of 12.74 units. The contribution of coordinate b* (yellowness) to the color of CPH was more relevant, probably due to its carotenoid content. Pico Hernández et al.^45^ reported a carotenoid content of 64.35 mg/g, using a supercritical fluid extraction system. Taking the correlation values into consideration, parameters L* and b* vs. moisture showed an negative relation (L* vs. moisture: r =  − 0.9512; p = 0.0000; R^2^ = 0.9049) and (b* vs. moisture: r =  − 0.9238; p = 0.0000; R^2^ = 0.8535), while the chromaticity parameter a* vs. moisture showed little or no correlation (a* vs. moisture: r =  − 0.1648; p = 0.5572; R^2^ = 0.0272).

With regard to the CBS, the clarity parameter (L*) ranged from 31.43 to 39.69 units (Table 5), whereas chromaticity parameters for a* and b* varied between 8.32 and 15.08 units and 6.14 and 16.78 units, respectively. As for the L* parameter, several authors reported a variation from 45 to 51 units^46, 47^. The variation in the L* parameter may be associated with the effect of temperature and drying times as a result of the process of caramelization of carbohydrates and amino acids, causing their browning and making their clarity decrease toward the darkest side. The results of chromaticity parameters, both a* and b*, were slightly similar to the results found in previous studies^39, 46, 47^.

### Methylxanthines and catechin derivatives of CPH and CBS

The results obtained from the LC-DAD analysis showing the content of theobromine, caffeine, and catechin derivatives are presented in Table 8. These compounds were previously identified in methanol extracts of the CPH and CBS^10, 13^. The theobromine content in CPH and CBS was 0.14 and 7.49 mg/g of sample, respectively. According to Nguyen and Nguyen^48^, the theobromine concentration in CPH obtained with various extraction systems, such as 70% chloroform and ethanol, was ~ 0.004 and ~ 0.02 mg/g, respectively, while under optimal extraction conditions with 70% ethanol for 90 min, it was ~ 0.07 mg/g. On the other hand, the theobromine content in CBS was found to be within the range established for toasted CBS of different geographic origins and genotypes, with values ranging from ~ 0.76 to ~ 9.03 mg/g^42^, although the values for CBS of different Mexican varieties ranged from 7.39 to 18.20 mg/g of sample^49^. The caffeine content in the CPH and CBS sample was 0.05 and 1.86 mg/g, respectively, which are lower than those reported by Botella-Martínez et al.^47^, who found that the caffeine content according to a particle size of 417–701 µm and < 417 µm was 11 mg/g and 6.13 mg/g, respectively^47^. The total methylxantine content in CPH and CBS was 0.19 and 9.35 mg/g per sample, respectively. Rojo-Poveda et al.^42^ reported similar values for CBS of Peruvian origin, with values between 8.3 and 9.7 mg/g.

**Table 8:** Content of methylxanthines and catechin derivatives obtained by optimal conditions of vacuum drying in cocoa pod husk and cocoa bean shell.

Compounds	Cocoa pod husk (mg/g)	Cocoa bean shell (mg/g)
Theobromine	0.14 ± 0.00	7.49 ± 0.01
caffeine	0.05 ± 0.00	1.86 ± 0.05
(+)-Catechin	1.23 ± 0.03	3.13 ± 0.09
(−)-Catechin 3-gallate	0.03 ± 0.00	0.61 ± 0.01
(−)-Epicatechin	2.23 ± 0.09	5.64 ± 0.03
(−)-Epicatechin-3-gallate	0.08 ± 0.00	0.53 ± 0.00
(−)-Epigallocatechin 3-gallate	1.60 ± 0.00	1.55 ± 0.02
(−)-Gallocatechin	n.d.	0.69 ± 0.03
(−)-Gallocatechin 3-gallate	0.17 ± 0.01	0.20 ± 0.01
Suma catechin + epicatechin	3.47	8.77
Suma catechin derivatives	1.87	3.58
Suma methylxanthines	0.19	9.35

Optimization conditions for vacuum drying: CPH (65 °C, 8 h and 75 mbar), and CBS (50 °C, 5 h and 100 mbar).

Regarding the catechin content of CPH and CBS, values ranging from 1.23 to 3.3 mg/g were found, with the values for CPH being similar to those reported by Valadez-Carmona et al.^50^, who reported values ranging between ~ 0.84 and ~ 2.28 mg/g for different dehydration systems, with microwave dehydration as the best treatment. Although the CBS catechin content was found to be within the range established by Hernández-Hernández et al.^49^, with values between 0.55 and 4.66 mg/g, the catechin content in toasted CBS of different geographical origins and genotypes was found to be lower, ranging from 0.012 to 0.18 mg/g^42^. Moreover, Botella-Martínez et al.^47^ reported a free catechin content ranging from 1.96 to 4.21 mg/g of sample.

Another bioactive component found in relevant amounts was epicatechin; in this study, CPH and CBS had epicatechin amounts of approximately 2.23 and 5.64 mg/g of sample. Furthermore, an investigation performed to assess the effect of microwaves drying, hot-air drying, and lyophilization on the epicatechin content of CPH resulted in values between 1.59 and 3.69 mg/g^50^. On the other hand, Rojo-Poveda et al.^42^ reported an epicatechin content of 0.044–0.74 mg/g in toasted CBS. These values were lower than those reported in the present study, although Hernández-Hernández et al.^49^ obtained much greater values, ranging from 4.40 to 26.68 mg/g of sample.

## Conclusion

RSM was used for optimizing the vacuum drying process of the CPH and CBS. ANOVA results provided evidence of the significance of the models for polyphenol and flavanol contents and RSA (p < 0.05). Although the lack-of-fit was nonsignificant (p > 0.05) and the correlation coefficient was greater than 0.9 for CPH, the ANOVA results proved that the models were nonsignificant (p > 0.05). The model for polyphenol content showed a lack-of-fit value (p = 0.046) with a contribution of 71%; the model for flavanol content showed a lack-of-fit value (p = 0.271) with a contribution of 44.9%; and that for RSA showed a lack-of-fit value (p = 0.826) with a contribution of 39.3% for CBS. The mathematical models generated for CPH were fit for experimental data, contrary to those generated for CBS, which showed nonfit values to predict responses.

## Data Availability

All data generated or analyzed during this study are included in this published article.

## References

[1] Chávez JAG, Baviera JMB, Pérez-Esteve É, Galanakis CM (2022). Valuation strategies for the biomass generated while producing and transforming cocoa into chocolate. Trends in Sustainable Chocolate Production.

[2] Soares TF, Oliveira MBPP (2022). Cocoa by-products: Characterization of bioactive compounds and beneficial health effects. Molecules.

[3] ICCO. *ICCO Quarterly Bulletin of Cocoa Statistics, Cocoa Year 2021/2022*. https://www.icco.org/statistics/#tab-id-6 (2022).

[4] Vergara-Mendoza M, Martínez GR, Blanco-Tirado C, Combariza MY (2022). Mass balance and compositional analysis of biomass outputs from cacao fruits. Molecules.

[5] Vásquez ZS (2019). Biotechnological approaches for cocoa waste management: A review. Waste Manag..

[6] Rojo-Poveda O, Barbosa-Pereira L, Zeppa G, Stévigny C (2020). Cocoa bean shell-a by-product with nutritional properties and biofunctional potential. Nutrients.

[7] Mendoza-Meneses CJ, Feregrino-Pérez AA, Gutiérrez-Antonio C (2021). Potential use of industrial cocoa waste in biofuel production. J. Chem..

[8] Mariatti F, Gunjević V, Boffa L, Cravotto G (2021). Process intensification technologies for the recovery of valuable compounds from cocoa by-products. Innov. Food Sci. Emerg. Technol..

[9] Belwal T (2022). Bioactive compounds from cocoa husk: Extraction, analysis and applications in food production chain. Foods.

[10] Cádiz-Gurrea ML (2020). LC-MS and spectrophotometric approaches for evaluation of bioactive compounds from Peru cocoa by-products for commercial applications. Molecules.

[11] Rojo-Poveda O (2021). Evaluation of cocoa bean shell antimicrobial activity: A tentative assay using a metabolomic approach for active compound identification. Planta Med..

[12] Rossin D (2021). Protective effect of cocoa bean shell against intestinal damage: An example of byproduct valorization. Antioxidants.

[13] Nguyen VT, Tran TG, Tran NL (2021). Phytochemical compound yield and antioxidant activity of cocoa pod husk (*Theobroma cacao* L.) as influenced by different dehydration conditions. Dry. Technol..

[14] Felice F (2020). Antioxidant effect of cocoa by-product and cherry polyphenol extracts: A comparative study. Antioxidants.

[15] Núñez-Sellés AJ, Abril-González AJ, Ramil-Mesa M (2021). PROMANCOA modular technology for the valorization of mango (*Mangifera indica* L.) and cocoa (*Theobroma cacao* L.) agricultural biowastes. Processes.

[16] Jing N (2021). Color sensory characteristics, nutritional components and antioxidant capacity of *Zanthoxylum bungeanum* Maxim. as affected by different drying methods. Ind. Crops Prod..

[17] Xu W (2021). Effects of different drying methods on sensory qualities and aroma compounds of finger citron (*Citrus medica* L. var. sarcodactylis Swingle) slices. J. Food Meas. Charact..

[18] Zhao R, Gao T (2015). Research progress of hot air-drying technology for fruits and vegetables. Adv. J. Food Sci. Technol..

[19] Valentina S, Savvas T, Bhat R (2022). The use of emerging dehydration technologies in developing sustainable food supply chain. Future Foods.

[20] Vega-Gálvez A (2021). Antimicrobial properties of papaya (*Vasconcellea pubescens*) subjected to low-temperature vacuum dehydration. Innov. Food Sci. Emerg. Technol..

[21] Souza da Costa B, Soldevilla Muro G, Oliván García M, Moltiva M-J (2022). Winemaking by-products as a source of phenolic compounds: Comparative study of dehydration processes. LWT Food Sci. Technol..

[22] Mella C (2022). Impact of vacuum drying on drying characteristics and functional properties of beetroot (*Beta vulgaris*). Appl. Food Res..

[23] Šumić Z, Tepić A, Vidović S, Jokić S, Malbaša R (2013). Optimization of frozen sour cherries vacuum drying process. Food Chem..

[24] Almeida-Trasviña F, Medina-Gonzalez S, Ortega-Rivas E, Salmerón-Ochoa I, Perez-Vega S (2014). Vacuum drying optimization and simulation as a preservation method of antioxidants in apple pomace. J. Food Process Eng..

[25] Šumić Z (2016). Modeling and optimization of red currants vacuum drying process by response surface methodology (RSM). Food Chem..

[26] Osama K, Mujtaba A, Siddiqui MH, Qadri OS, Younis K (2022). Optimization of vacuum drying and determination of functional properties of Kadam (*Neolamarckia cadamba*) fruit powder. J. Food Process Preserv..

[27] Jaimez RE (2022). *Theobroma cacao* L. cultivar CCN 51: A comprehensive review on origin, genetics, sensory properties, production dynamics, and physiological aspects. PeerJ.

[28] Prior RL, Wu X, Schaich K (2005). Standardized methods for the determination of antioxidant capacity and phenolics in foods and dietary supplements. J. Agric. Food Chem..

[29] Kupina S, Fields C, Roman MC, Brunelle SL (2018). Determination of total phenolic content using the Folin-C assay: Single-laboratory validation, first action 2017.13. J. AOAC Int..

[30] Ramos-Escudero F (2021). Colour, fatty acids, bioactive compounds, and total antioxidant capacity in commercial cocoa beans (*Theobroma cacao* L.). LWT Food Sci. Technol..

[31] Gallego AM (2018). Transcriptomic analyses of cacao cell suspensions in light and dark provide target genes for controlled flavonoid production. Sci. Rep..

[32] Brand-Williams W, Cuvelier ME, Berset C (1995). Use of a free radical method to evaluate antioxidant activity. LWT Food Sci. Technol..

[33] Wang H, Provan GJ, Helliwell K (2003). HPLC determination of catechins in tea leaves and tea extracts using relative response factors. Food Chem..

[34] Breig SJM, Luti KJK (2021). Response surface methodology: A review on its applications and challenges in microbial cultures. Mater. Today Proc..

[35] Leyva-Jiménez FJ (2022). Application of response surface methodologies to optimize high-added value products developments: Cosmetic formulations as an example. Antioxidants.

[36] Che Sulaiman IS (2017). Effects of temperature, time, and solvent ratio on the extraction of phenolic compounds and the anti-radical activity of *Clinacanthus nutans* lindau leaves by response surface methodology. Chem. Cent. J..

[37] Vakula A (2020). Vacuum drying of sweet cherry: Artificial neural networks approach in process optimization. J. Food Process Preserv..

[38] Rebollo-Hernanz M (2021). Extraction of phenolic compounds from cocoa shell: Modeling using response surface methodology and artificial neural networks. Sep. Purif. Technol..

[39] Delgado-Ospina J (2021). Bioactive compounds and techno-functional properties of high-fiber co-products of the cacao agro-industrial chain. Heliyon.

[40] Campos-Vega R, Nieto-Figueroa KH, Dave Oomah B (2018). Cocoa (*Theobroma cacao* L.) pod husk: Renewable source of bioactive compounds. Trends Food Sci. Technol..

[41] Indrianingsih AW (2021). In vitro studies of antioxidant, antidiabetic, and antibacterial activities of *Theobroma cacao*, *Anonna muricata* and *Clitoria ternatea*. Biocatal. Agric. Biotechnol..

[42] Rojo-Poveda O, Zeppa G, Ferrocino I, Stévigny C, Barbosa-Pereira L (2021). Chemometric classification of cocoa bean shells based on their polyphenolic profile determined by RP-HPLC-PDA analysis and spectrophotometric assays. Antioxidants.

[43] Barbosa-Pereira L, Belviso S, Ferrocino I, Rojo-Poveda O, Zeppa G (2021). Characterization and classification of cocoa bean shells from different regions of Venezuela using HPLC-PDA-MS/MS and spectrophotometric techniques coupled to chemometric analysis. Foods.

[44] Delgado-Ospina J (2021). Cacao pod husk flour as an ingredient for reformulating frankfurters: Effects on quality properties. Foods.

[45] Pico Hernández SM, Jaimes Estévez J, López Giraldo LJ, Murillo Méndez CJ (2019). Supercritical extraction of bioactive compounds from cocoa husk: Study of the main parameters. Rev. Fac. Ing..

[46] Fakhlaei R, Rozzamri A, Hussain N (2020). Composition, color and antioxidant properties of cocoa shell at different roasting temperatures. Food Res..

[47] Botella-Martínez C (2021). Ghanaian Cocoa (*Theobroma cacao* L.) bean shells coproducts: Effect of particle size on chemical composition, bioactive compound content and antioxidant activity. Agronomy.

[48] Nguyen VT, Nguyen NH (2017). Proximate composition, extraction, and purification of theobromine from cacao pod husk (*Theobroma cacao* L.). Technologies.

[49] Hernández-Hernández C (2019). Cocoa bean husk: Industrial source of antioxidant phenolic extract. J. Sci. Food Agric..

[50] Valadez-Carmona L (2017). Effects of microwaves, hot air and freeze-drying on the phenolic compounds, antioxidant capacity, enzyme activity and microstructure of cacao pod husks (*Theobroma cacao* L.). Innov. Food Sci. Emerg. Technol..

